# Compressive Buckling Fabrication of 3D Cell‐Laden Microstructures

**DOI:** 10.1002/advs.202101027

**Published:** 2021-07-15

**Authors:** Zhaowei Chen, Nanditha Anandakrishnan, Ying Xu, Ruogang Zhao

**Affiliations:** ^1^ Department of Biomedical Engineering State University of New York at Buffalo Buffalo NY 14260 USA

**Keywords:** cell‐laden microstructures, compressive buckling, engineered tissue, polymeric biomaterials, toughness

## Abstract

Tissue architecture is a prerequisite for its biological functions. Recapitulating the three‐dimensional (3D) tissue structure represents one of the biggest challenges in tissue engineering. Two‐dimensional (2D) tissue fabrication methods are currently in the main stage for tissue engineering and disease modeling. However, due to their planar nature, the created models only represent very limited out‐of‐plane tissue structure. Here compressive buckling principle is harnessed to create 3D biomimetic cell‐laden microstructures from microfabricated planar patterns. This method allows out‐of‐plane delivery of cells and extracellular matrix patterns with high spatial precision. As a proof of principle, a variety of polymeric 3D miniature structures including a box, an octopus, a pyramid, and continuous waves are fabricated. A mineralized bone tissue model with spatially distributed cell‐laden lacunae structures is fabricated to demonstrate the fabrication power of the method. It is expected that this novel approach will help to significantly expand the utility of the established 2D fabrication techniques for 3D tissue fabrication. Given the widespread of 2D fabrication methods in biomedical research and the high demand for biomimetic 3D structures, this method is expected to bridge the gap between 2D and 3D tissue fabrication and open up new possibilities in tissue engineering and regenerative medicine.

## Graphical abstract

This work represents the first study to explore the possibility of using compressive buckling‐based fabrication technology to build 3D cell‐laden microstructures from originally 2D planar structures. It is expected that this novel approach will help to significantly expand the utility of the well‐established 2D fabrication techniques and open up new possibilities in tissue engineering and regenerative medicine.

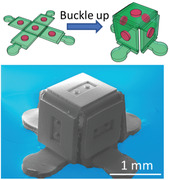

## 1 Introduction

Native tissue is composed of multiple types of cells and extracellular matrix (ECM) that are organized into complex spatial patterns. This highly specialized architecture of living tissues is critical for their biological functions during tissue homeostasis, regeneration, and embryonic development.^[^ ^1^, ^2^, ^3^ ^]^ Engineered tissue models recapitulating the three‐dimensional (3D) and hierarchical structure of the native tissue are valuable tools for the study of tissues’ biological functions, but their fabrication represents one of the biggest challenges in tissue engineering.^[^ ^4^, ^5^, ^6^, ^7^ ^]^ Lithography‐based techniques such as microfluidic channels and microtissue patterning have been developed in recent years and have become the workhorse in tissue engineering for the creation of biomimetic tissue structures.^[^ ^8^, ^9^, ^10^, ^11^ ^]^ Microfluidic‐based organ‐on‐chip systems are currently at the main stage for the modeling of diseases and the preclinical testing of a variety of drugs.^[^ ^6^, ^12^, ^13^ ^]^ However, despite their success and widespread use, lithography‐generated biomimetic structures are predominantly two‐dimensional (2D) and only represent very limited out‐of‐plane tissue structure.^[^ ^14^, ^15^ ^]^ Polymeric 3D structures with substantial thickness and vasculature‐like channels have been created by stacking 2D patterned layers, but this fabrication process is slow and labor and resource‐intensive.^[^ ^16^ ^]^

Recently, 3D mesoscale silicon structures have been created through compressive buckling of 2D patterns made through planar lithography methods.^[^ ^17^, ^18^ ^]^ In this approach, compressive force was applied to the slender 2D silicon elements through spatially patterned binding sites, causing the silicon element to buckle. The out‐of‐plane displacement occurred during the buckling process allows the rapid formation of a 3D structure with a substantial height. This method enables geometric transformation from 2D mesostructures to extended 3D structures, which can be used for amplifying electrical signals, soft electronics, and electronic stimulators.^[^ ^19^, ^20^, ^21^ ^]^ This method holds great potential to make next‐generation wearable devices and electronic skin that can be applied for healthcare monitoring. However, it has not been used in the fabrication of biomimetic tissue structures, especially cell‐laden structures.

In the current study, we harnessed compressive‐buckling principle to allow the rapid formation of 3D biomimetic polymeric structures from 2D patterns created using lithography‐based methods. We identified mechanical properties of the polymer that are critical to the compressive‐buckling process and developed sucrose‐based sacrificial fabrication method to allow cell seeding onto compression‐buckled 3D polymeric structures. Using this method, we achieved the out‐of‐plane delivery and deposition of cell and ECM patterns with high spatial precision. As a proof of principle, we demonstrate the fabrication of a cell‐laden osteon structure using this novel approach. Our results show that this novel, cell culture compatible approach allows rapid transformation of planar patterns created using lithography‐based methods to 3D biomimetic structures. It is expected that this novel approach will help to significantly expand the utility of the established 2D fabrication techniques for 3D tissue fabrication. Given the widespread of 2D fabrication methods in biomedical research and the high demand for biomimetic 3D structures, the presented novel approach is expected to bridge the gap between 2D and 3D tissue fabrication and open up new possibilities in tissue engineering and regenerative medicine.

## 2 Results

### 2.1 Process of Compressive Buckling of Cell‐Laden 2D Precursor Patterns to Form 3D Mesoscale Structures

**Figure 1:**
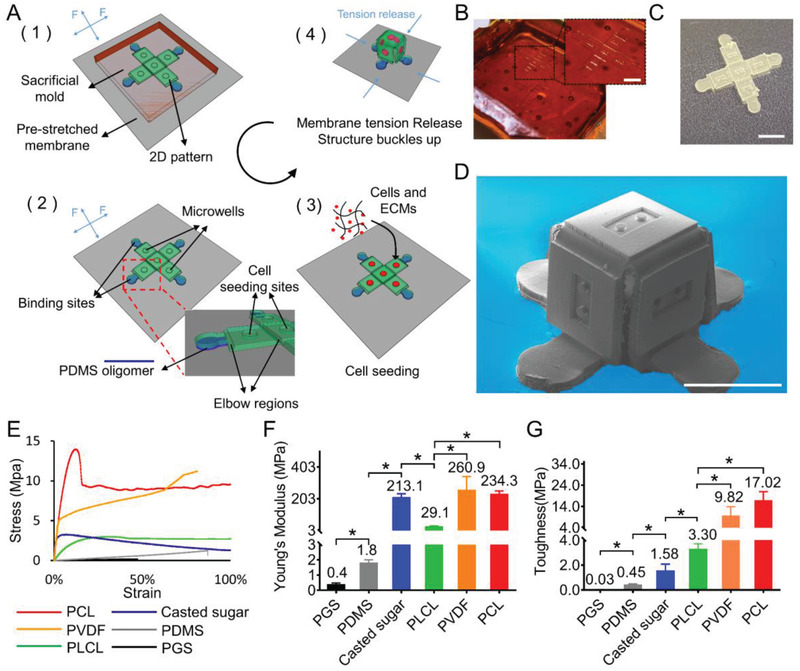
Overview of the compressive buckling fabrication process and material characterization. A) Schematic overview of the fabrication process. 1) 2D polymer pattern is cast in a sacrificial sugar mold and is bound to a pre‐stretched silicone membrane at designed binding sites. 2) 2D pattern is released from the sugar mold. 3) Cell‐laden hydrogel prepolymer is seeded in the microwells in the 2D pattern and is maintained in cell culture. 4) 2D pattern buckled up to a 3D cell‐laden microstructure via the release of pre‐tension in the membrane. B) A sacrificial sugar mold with the 2D pattern for a box design. Scale bar is 2 mm. C) A 2D polymeric pattern released from the sugar mold. Scale bar = 2 mm. D) SEM image of a box design formed through compressive buckling. Note two‐pillar microwells on every faceplate. Scale bar is 1 mm. E) Representative stress‐strain curves, F) Young's modulus, and G) Toughness of the polymeric materials tested for compressive buckling (*n* = 3–5 for each material). *p < 0.05 by ANOVA using GraphPad Prism (GraphPad Software). Data are presented as mean ± SD.

Based on the compressive buckling principle used for the semiconductor materials,^[^ ^17^ ^]^ we developed a cell‐culture compatible fabrication process for the compressive buckling of cell‐laden 2D precursor patterns, as shown in **Figure** **1A**, and the side view of some key steps are shown in Figure S1, Supporting Information. First, a 2D pattern was fabricated by SU‐8 photolithography and then transferred to a PDMS mold through soft lithography (Figure S2, Supporting Information). This pattern contains several faceplates connected by strategically placed elbow regions with reduced thickness. Microwell arrays were created in the faceplates to hold cell aggregates in 3D space, and the elbow regions allow bending of the material during structural buckling. The pattern on PDMS was transferred to a sacrificial sugar mold with high fidelity (Figure 1A (1) and Figure S2, Supporting Information). Prepolymer such as Polylactide‐co‐caprolactone (PLCL, LA: CL = 30: 70) was then poured into the sugar mold and allowed to cure (Figures S1 and S2, Supporting Information). The advantage of using sugar mold is that it does not react with the organic solvent used to prepare the prepolymer, thus allowing the transfer of patterns with high geometrical fidelity. The cured polymer pattern was bound to a pre‐stretched silicone membrane at pre‐defined binding sites through PDMS oligomer microcontact printing,^[^ ^22^ ^]^ and then released from the sugar mold by dissolving the sugar mold in water (Figure 1A (2)). Cell‐laden hydrogel prepolymer was seeded in the microwells in the formed 2D mesostructure, which was then maintained in cell culture to allow individual microtissue formation (Figure 1A (3)). After the microtissues form, compressive forces generated via the release of pre‐tension in the silicone membrane were applied to the 2D pattern through the binding sites, causing the 2D pattern to buckle into a 3D cell‐laden mesoscale structure (Figure 1A (4)). Figure 1B,C shows a representative sacrificial sugar mold and a released 2D pattern. Figure 1D shows a representative 3D polymeric box structure formed through compressive buckling with an edge length of 1000 µm. There is a rectangular, 600 µm long microwell on each of the five faceplates.

### 2.2 Toughness is a Critical Biomaterial Property for Fabrication using Compressive Buckling

To understand the compatibility of compressive buckling method with polymeric materials, we performed mechanical testing on casted polymer samples and determined the mechanical parameters that are critical for compressive buckling process. We found that for the compressive buckling to occur, a material needs to have both good Young's modulus and ductility. This can be measured by the toughness of the material, as calculated by the area below the stress–strain curve. The mechanical properties including stress–strain curves, Young's modulus, and toughness of several commonly used bio‐compatible polymers were measured and their suitability for compressive buckling was compared (Figure 1E–G and Table S1, Supporting Information). While PDMS has good ductility, its Young's modulus is not high enough, resulting in low toughness that prevents compressive buckling. Similarly, poly(glycerol sebacate) (PGS) is too soft, making it unsuitable for compressive buckling.^[^ ^23^ ^]^ In contrast, the sugar polymer's Young's modulus is high, but its poor ductility (brittle) results in low toughness. Poly(lactic‐co‐glycolic acid) (PLGA, PLA:PGA = 50:50) also suffers from poor ductility that prevented compressive buckling (Figure S3, Supporting Information). The toughness of all the above materials is below 3 MPa. Both polycaprolactone (PCL, 45–55 kDa) and its copolymer Poly(lactide‐co‐caprolactone) (PLCL, 45–55 kDa) with PLA to PCL ratio of 3:7 were shown to support the compressive buckling process. Their Young's modulus and ductility are both reasonably high, resulting in high toughness of 17.02 and 3.3 MPa respectively. Since PLCL has improved degradability than PCL, which is potentially useful in many biomedical applications, we chose PLCL in our later studies. At higher PLA to PCL ratios of 5:5 and 6:4, the increased Poly(lactide) component caused reduction in the PLCL Young's modulus, making it unsuitable for compressive buckling. PVDF has similar mechanical properties as the PCL, making it a suitable material for compressive buckling (Figure 1E–G and Figure S4, Supporting Information). To demonstrate the impact of material properties on the buckling performance of different materials, we performed finite element analysis of the buckling process of a simple cross‐shaped 2D structure made of either soft (PDMS) or tough (PLCL) polymers. We showed that the structure made of soft material has a much lower critical buckling force (eigenvalue) than the structure made of tough material, suggesting that pre‐mature buckling (failure) will occur in soft material (Figure S5, Supporting Information).

### 2.3 Compressive Buckling Formation of Various 3D Polymeric Structures

**Figure 2:**
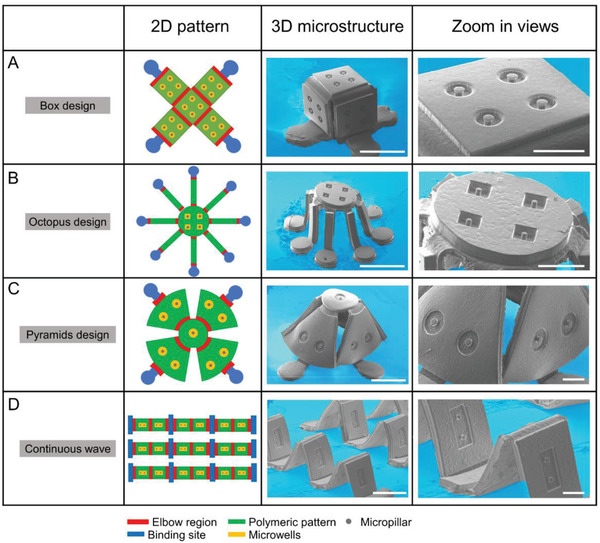
Representative 2D pattern designs and compressive buckling formed 3D microstructures. A) A box design with four circular 100 µm diameter microwells on each faceplate. A center micropillar is in each microwell. The 3D box structure is formed through releasing of a 300% pre‐stretch. B) An octopus design with eight high aspect‐ratio legs. Four square 100 µm size microwells are created on the top faceplate. A pre‐stretch of 400% is used in the formation of this 3D structure. C) A pyramid design with two circular microwells on the side faceplates and one circular microwell on the top faceplate. A pre‐stretch of 200% is used to form this structure. D) A continuous‐wave design with a rectangular microwell on each faceplate. A pre‐stretch of 200% is used to form this structure. Scale bar for 3D structure view is 1 mm and scale bar for zoom‐in view is 300 µm.

To demonstrate the fabrication power of the compressive buckling method and its compatibility with different 3D mesoscale structures, we designed and fabricated several representative 3D structures. **Figure** **2** shows the schematic of 2D designs, the SEM image of the corresponding 3D structures after buckling and the zoom‐in view showing the structural details for the box (Figure 2A), octopus (Figure 2B), pyramid (Figure 2C), and continuous‐wave (Figure 2D) designs. The location of the important structural components including the binding site, the elbow region, and the microwells for microtissue seeding are illustrated in the 2D design. The amount of pre‐stretch needed for each design was calculated and tested in the compressive buckling process to ensure the formation of desired 3D structure. As shown by the SEM images, the high quality of the formed 3D structures, such as the perpendicular surfaces of the box structure, the equal height of the legs in the octopus structure, and the equal space and bending angles in the continuous wave structure, demonstrates the power of the compressive buckling method for the precision control of the 3D arrangement of the structural elements. Furthermore, the smooth surface of the 3D structures and the well‐retained micro‐structural features including the microwell arrays and the micropillars in the microwells suggest that the developed fabrication process can allow high fidelity transformation of the 2D planar patterns into 3D.

### 2.4 Compressive Buckling for Spatially‐Controlled Delivery of Cells and Tissues in 3D

**Figure 3:**
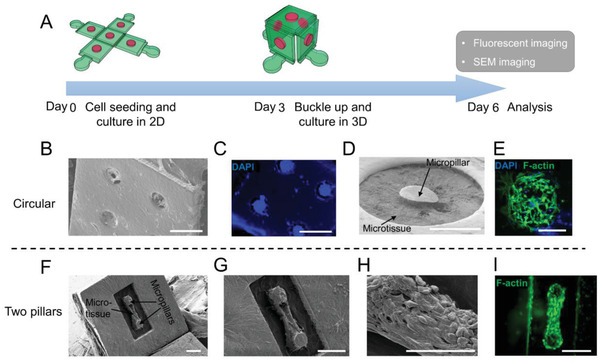
Representative images of cell and ECM‐laden 3D microstructures. A) A schematic shows the formation of cell and ECM‐laden 3D microstructures. B) SEM image and C) DAPI staining image of a faceplate in a 3D box structure. Cells and collagen are encapsulated in the microwells. D) SEM image of the microtissue formed in a single circular microwell. Cells are embedded in the collagen matrix. E) F‐actin and DAPI staining of the microtissue in the circular microwell. Note cells spread very well in the collagen matrix. Scale bars for B‐E are 200 µm. F) SEM image and G) zoom‐in view of a dog‐bone shaped microtissue formed in a rectangular microwell on a titled faceplate of a continuous wave structure. The microtissue formed through cell contraction‐mediated tissue self‐assembly. The two micropillars serve as boundary conditions to guide the formation of the microtissue. H) Zoom‐in view of a portion of the dog‐bone shaped microtissue shows cell spreading on collagen matrix. I) F‐actin staining of the dog‐bone shaped microtissue. Scale bars for (F–I) are 150 µm.

Next, we examined the compatibility of the compressive buckling method with cell seeding and culture. Cell‐laden collagen matrix was seeded in the microwells of the 2D precursor patterns through centrifugation and cultured for 3 days. The 2D precursor patterns were then buckled up to form 3D structures, which were cultured for another 3 days (**Figure** **3A**). Figure 3B–I showed the SEM and fluorescence images of microtissues formed in circular microwells on the vertical wall of a box structure and in rectangular microwells on the tilted wall of a continuous wave structure. Microtissues were able to fill multiple circular microwells in the box structure (Figure 3B), and a single microtissue occupied a substantially large area in the continuous wave structure (Figure 3F). In a single circular microwell, embedded fibroblasts self‐organized the collagen matrix around the center micropillar and the formed microtissue distributed quite uniformly in the microwell (Figure 3C–E). In the rectangular microwell, fibroblasts self‐organized and compact the collagen matrix around the two micropillars, leading to the formation of a dog‐bone shaped microtissue anchored to the micropillars. Due to the mechanical constraints provided by the micropillars, cells aligned along the longitudinal axis of the microtissue (Figure 3G–I). In both the circular and dog‐bone shaped microtissues, the morphology of microtissues formed in the 2D precursor pattern was not disrupted by the compressive buckling process. Microtissue morphology and viability are maintained in the subsequent culture in the 3D structure. The morphology of dog‐bone shaped microtissue cultured in the 3D structure at day 6 was not different from those cultured in 2D device for 6 days (Figure S6, Supporting Information). Together, these results show that the compressive buckling process is compatible with the seeding and culture of 3D cell‐laden structures with complex morphology and structural details, and it enables spatially‐controlled delivery of cells and microtissues into the 3D space.

### 2.5 Demonstration of Bone Tissue Engineering Using Compressive Buckling

**Figure 4:**
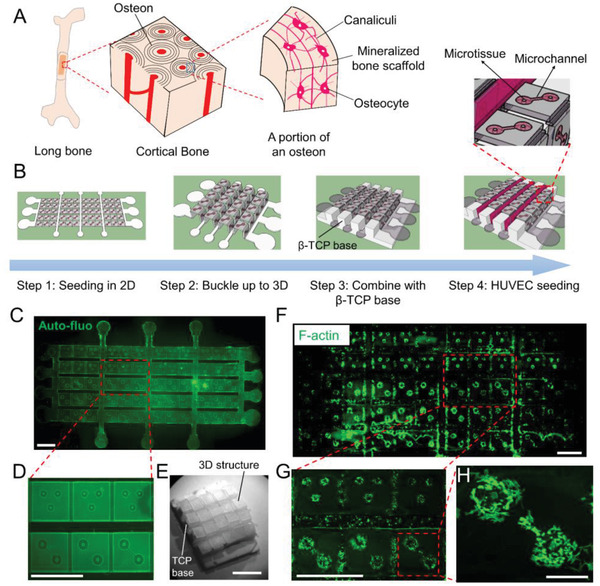
A bone osteon tissue model formed through compressive buckling. A) Schematic shows the hierarchical structure of bone and osteon. B) Schematic shows the fabrication steps for the osteon tissue model. Microwells and interconnecting microchannels are patterned on the surface of buckled 3D structure to mimic the lacunae and canaliculi (inset). 3D structure is combined with a pre‐casted *β*‐TCP base that mimics the mineral content of the bone tissue and provides loading bearing capacity. C) Auto fluorescence image of the 2D pattern. D) Enlarged view shows three‐well and two‐well lacunae structures. The circular microwells are connected by the microchannels. E) Bright‐field image shows the assembled bone tissue model scaffold without cells. Scale bar is 0.5 cm. F) F‐actin staining of hMSCs seeded in the 2D pattern. G) Enlarged view shows cell populated lacunae structures. Scale bar is 2 mm in panel (C–F). H) Enlarged view of a two‐well lacunae structure. Cells in a circular well not only spread well but also migrate toward the other well via the microchannel. Scale bar is 300 µm.

To demonstrate the utility of the compressive buckling method for tissue engineering, we created an osteon‐like structure. Osteon is the basic building unit of bone tissue and is characterized by osteocytes sparsely distributed in the mineral bone scaffold. Each osteocyte is contained in a small cavity, known as lacunae, and different osteocytes are connected through canaliculi which are small channels in the bone scaffold (**Figure** **4A**).^[^ ^24^ ^]^ Figure 4B and Figure S7A, Supporting Information, show the schematic of the fabrication process for the osteon‐like structure. Through compressive buckling of a 2D precursor design, multiple 3D ridge structures were formed. Each of the ridge structure contains multiple repeating “n” shaped units (Figure S7B, Supporting Information). Microwells and interconnecting microchannels were patterned on three sides of the “n” units to mimic the lacunae and canaliculi. Human mesenchymal stem cell (hMSC)‐populated microtissues were grown and differentiated in the microwells to mimic the osteocytes encapsulated in the ECM. To provide load‐bearing capacity to the structure, patterned ridges made of mixed *β*‐tricalcium phosphate (*β*‐TCP) and PCL were inserted underneath the buckled structure. Finally, human umbilical vein endothelial cell (HUVEC)‐laden Gelatin Methacrylate (GelMA) gels were seeded in the gaps between the ridges to mimic the blood vessels. Fluorescence images of the 2D precursor design show that two‐microwell and three‐microwell patterns were created on the pattern surface to mimic the lacunae that contain the osteocytes. Micro‐channels of 30 µm width were formed to connect between the microwells, mimicking the canaliculi (Figure 4C,D). After buckling, the 3D ridge structure fits well to the *β*‐TCP base (Figure 4E). F‐actin staining shows that hMSC populated microtissues grew well in the microwells of the 2D pattern (Figure 4F). Zoom‐in view of a two‐microwell pattern shows hMSCs well spread in the collagen matrix and some hMSCs migrating along the microchannel (Figure 4G,H). After 3 days’ culture in 2D, pattern was buckled up to form the osteon‐like structure, which is about 1.5 cm × 1.5 cm with a thickness around 0.3 cm.

**Figure 5:**
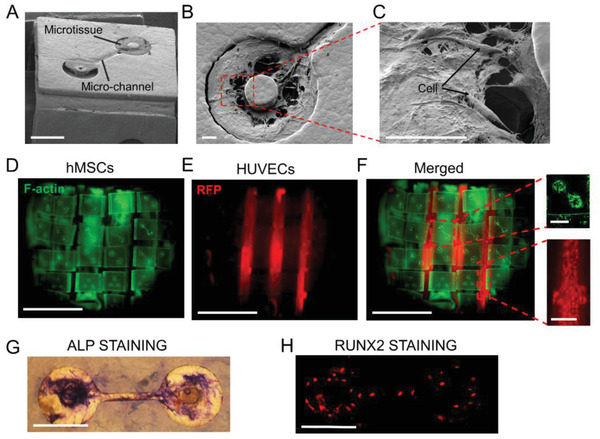
Characterization of the engineered bone tissue model. A) SEM image of an hMSC‐microtissue populated, two‐well lacunae structure on the top faceplate of the 3D structure. Scale bar is 400 µm. B) SEM image of the microtissue in one of the microwells in the lacunae structure. C) Enlarged view of the cells and collagen matrix in the microwell. Scale bar is 50 µm in panel (B–C). D–E) Fluorescent images of the hMSCs (green, D) and HUVECs (red, E) in the bone tissue model. F) Merged fluorescent image shows well‐controlled spatial distribution of different cell types. Scale bar for large views is 5 mm, zoom‐in images are 500 µm. G) ALP staining shows osteogenic differentiation of the hMSCs in the microtissue. Scale bar is 300 µm. H) Runt‐related transcription factor 2 (RUNX2) staining of hMSCs in the microtissue under osteogenic differentiation. Scale bar is 300 µm.

The morphology of the patterned microtissues was maintained well after buckling (**Figure** **5A**–C). The 3D construct was maintained in osteogenic differentiation media for 3 weeks. After 3 weeks of osteogenic differentiation, we fitted the 3D cell‐laden construct with the TCP‐PCL substrate and seeded HUVEC and hMSC‐laden (10:1 ratio) GelMA into the gaps between the ridges. The final assembled construct contains approximately 70% mineral content and 30% protein content, which is consistent with the human bone composition.^[^ ^24^ ^]^ Fluorescence staining of the whole construct shows well‐segregated tissue areas (green ‐ hMSCs in microwells; red ‐ HUVECs in the gaps) (Figure 5D–F). Compression test of the construct shows the mechanical modulus to be 67 ± 8 MPa, which is consistent with the value of other engineered bone construct using similar mineral materials (Figure S8A, Supporting Information).^[^ ^25^ ^]^ SEM images show porous cross‐section of the TCP/PCL construct (Figure S8B, Supporting Information). Alkaline Phosphatase (ALP) and Runt‐related transcription factor 2 (RUNX2) staining show substantial level of osteogenic differentiation of the embedded hMSCs, suggesting that compressive buckling‐formed 3D structures are compatible with relatively long‐term culture required for the differentiation and maturation of certain types of engineered tissues (Figure 5G,H).

## 3 Discussion

The hierarchical 3D tissue architecture is important to tissue's biological functions.^[^ ^26^, ^27^, ^28^, ^29^ ^]^ Recreating the native tissue structure in engineered tissue is a challenging task and a variety of approaches such as microfabrication and 3D printing have been developed in the past decades.^[^ ^6^, ^30^, ^31^, ^32^, ^33^ ^]^ 3D bioprinting is an emerging technology that allows spatially‐controlled deposition of cells and ECMs and has been used to build engineered tissues representing bone,^[^ ^34^ ^]^ vasculature,^[^ ^16^, ^35^ ^]^ liver,^[^ ^36^ ^]^ and cartilage.^[^ ^37^ ^]^ However, extrusion and stereolithography‐based 3D bioprinting approaches still suffer from relatively low spatial resolution, slow printing speed that can affect the part quality and cell viability, and limited choice of printable bioinks. Two‐photon bioprinting improves the spatial resolution, but suffers from slow printing speed that limits its ability to fabricate large‐size engineered tissues.^[^ ^38^ ^]^ While 3D bioprinters are increasingly affordable these days, they are still inaccessible by many researchers and clinicians in the biomedical field. In contrast, microfabrication methods such as photolithography and soft lithography have been well developed in the past two decades and their applications such as microfluidics have widespread into essentially every area of the biomedical research. In the current work, through combining compressive buckling with 2D microfabrication, we create 3D polymeric cell‐laden scaffolds with high spatial resolution, thus opening up new avenues for the application of 2D methods in the fabrication of 3D multiscale tissues. Given the widespread of 2D microfabrication in the biomedical research community, this new approach is expected to significantly broaden the utility of the 2D fabrication methods in the era of 3D tissue engineering.

The transformation of patterned 2D polymer layers into 3D structures has been previously achieved through self‐folding of the 2D layers. This is achieved through the differential swelling of the environmental‐sensitive polymer bi‐layer under temperature or pH stimuli.^[^ ^39^, ^40^, ^41^ ^]^ This approach has been used in the formation of self‐deployable 3D miniature tubes or cubes for drug delivery and the fabrication of certain electronics devices.^[^ ^40^, ^42^, ^43^ ^]^ However, while this method is rapid and simple, it does not offer much spatial control on the formed 3D structure and the environmental stimuli such as the changing of temperature and pH are not compatible with cell culture. In contrast, the compressive buckling approach presented in the current study allows spatially‐controlled formation of 3D structure and the entire fabrication process is cell culture compatible, thus having much higher application potential in tissue engineering and regenerative medicine.

The use of sacrificial sugar mold is critical to the entire compressive buckling fabrication process. Sugar has been used as a sacrificial material for tissue fabrication in previous studies. Miller et al. used 3D printed sugar lattice as a sacrificial material to build patterned vascular networks for the application of tissue engineering.^[^ ^35^ ^]^ Moraes et al. used sugar mold as a sacrificial material to release the supersoft PDMS patterns, which can not be fabricated using conventional demolding method.^[^ ^44^ ^]^ Several unique features of the sugar mold are particularly helpful to the fabrication process presented in the current study, including its ability to allow high fidelity (≈20 µm) transfer of super high aspect ratio structures and its high dissolvability in water and very low dissolvability in organic solvent. The differential dissolvability of the sugar mold allows the molding of biocompatible polymers that were dissolved in organic solvent and the release of such as polymer patterns in water while maintaining their geometrical fidelity. This can not be achieved using conventional PDMS‐based molding and demolding techniques.

Sufficient toughness is another critical factor for the successful compressive buckling of polymeric structures. The key to toughness is a good combination of Young's modulus and ductility. We performed material optimization to seek polymer materials with both suitable mechanical properties and biocompatibility. We showed that PLCL, with both good toughness and biocompatibility, is a suitable choice for the compressive buckling fabrication process. PLCL (30:70) formed 3D structures can achieve an aspect ratio as high as 30:1 without collapsing. Similar co‐polymers and polymer mixtures such as PLGA‐PCL‐PLGA with a toughness larger than 3 MPa are expected to have similar compressive buckling performance.^[^ ^45^ ^]^ PVDF (polyvinylidene fluoride), a polymer with great piezoelectric properties, was found to have sufficient Young's modulus and toughness needed for compressive buckling (Figure S4, Supporting Information).^[^ ^46^ ^]^ In contrast, PLGA (50:50) alone has high mechanical modulus but low ductility, making it too brittle to undergo the buckling process (Figure S3, Supporting Information).^[^ ^47^ ^]^ Based on the material characterization performed in the current study, a toughness higher than 3 MPa seems to be sufficient for the compressive buckling process. Future strategies to discover suitable materials for compressive buckling may involve blending of biocompatible high modulus polymers with ductile polymers. With suitable materials, it is expected that this novel approach can be broadly applied to many different tissue types, thus improving our ability to fabricate 3D tissue models for disease modeling and drug screening. Exploring the utility of this novel fabrication method for tissue engineering could be a promising direction for future research.

## 4 Experimental Section

### PDMS Mold Fabrication

The PDMS (Dow Corning, Sylgard 184) mold was made by multilayer microlithography and softlithography techniques as previously described.^[^ ^48^, ^49^, ^50^ ^]^ Briefly, two layers of SU‐8 were successively deposited on the silicon wafer (University Wafer), exposed to UV light through transparency masks printed by laser plotting (CAD/Art Services Inc.), and baked and developed according to manufacturer's protocols using an OAI maskaligner with a U‐360 band pass filter. Pattern with the folding sites and microwells on the SU‐8 wafer was then transferred to PDMS mold made with 10:1 ratio of dimer to curing agent via replica molding. The thickness of each layer was optimized to be around 100 µm for both to make sure buckling happened only at folding sites. One or two micropillars were designed in the microwell to hold the cultured tissue inside.

### Sacrificial Sugar Mold Fabrication

1:1 (w/w) ratio of Light corn syrup (Karo, Walmart) and Pure cane sugar (Domino Sugar, Walmart) was well mixed and microwave heated until the mixture turned to yellow liquid state with suitable viscosity. Then the hot liquid sugar was poured into a preheated PDMS molds (pre‐heated on a 130 °C hot plate for 5 min) until fully cover whole patterns. Air baubles trapped inside of the PDMS mold were degassed by a 15 PSI pump (VacuMaster) until no baubles were found.^[^ ^44^ ^]^ Second time degassing was needed if major bobbles were still obvious. The final sugar mold was peeled off from PDMS mold after fully cooled down to room temperature and stored in a vacuum chamber for later use.

### Mechanical Tests for Biomaterials of Interest

The mechanical properties of the biomaterials of interest were using a motorized uniaxial testing system (Mark10) fitted with an M5‐012 digital force gauge with measurement resolution of 500 mN. Five specimens were tested for each material. For PDMS and PGS (Secant Medical) specimens, 1* 5 mm^2^ square cross‐section and 10 mm in length were tested. The casted sugar for mechanical tests was the sacrificial sugar mold material supplemented with gluten and starch to increase the deformability. For PLCL (PolySciTech, AP034, AP074, AP067, AP142), PCL (Sigma, 440 744), and casted sugar specimens, 0.2*3 mm^2^ square cross‐section and 10 mm in length were tested. High‐strength PVDF (McMaster‐Carr) film was cut into strips with the dimension of 0.076 mm (thickness)* 1 mm (width)* 20 mm (length). PLGA (50: 50, PolySciTech) was casted into strips with the dimension of 0.8 mm (thickness)* 0.5 mm (width)* 24 mm (length). All specimens were mechanically tested at a speed of 0.1 mm s^−1^ for up to 200% strain unless broken. The stress values were determined as the loading values divided by the initial cross‐sectional area of each test specimen. The strain values were determined as the deformation values divided by the initial specimen length. For all the specimens, the elastic modulus was calculated as the slope of the initial linear portion of the stress–strain curve, the toughness was calculated as the area below the stress‐strain curve. For demonstration purpose, strip samples were hand compressed by two PDMS blocks until reaching around 50% of their initial length or breaking.

### Compressive Buckling Based Fabrication Using PLCL

PLCL (poly lactide‐co‐caprolactone, LA:CL = 30:70, ester endcap with molecular weight 85–100 Kda) polymer was dissolved in 1,4‐Dioxane (TCI AMERICA, D0860) at the concentration of 20% (w/v) and filled in the sugar mold. After PLCL was fully dried in a desiccator with desiccant for at least 2 days, extra polymer material was removed by a razor blade with the help of a small droplet of dioxane to dissolve PLCL on the surface (Figure S1, step 2–3, Figure S2, step 4, Supporting Information). After fully dried overnight, the extra thin membrane of PLCL layer left on top was removed by gently polishing the surface with ultra‐fine sandpaper. Then a PDMS oligomer microcontact printing method,^[^ ^22^ ^]^ or PR‐1205 prime coat (Dow Corning, 4094883) microcontact printing method, was used to bind the PLCL with a pre‐stretched silicone membrane (Specialty Manufacturing, Saginaw, MI) at specific binding sites (Figure 1A (1), Figure S1, step 4, Figure S2, step 5–6, Supporting Information). The pre‐stretched silicone membrane was fixed on a custom‐made stretch device (Figure S9, Supporting Information) that we can change the stretch degree by changing the membrane size and clip mounting position before stretch. After that, the sacrificial sugar mold was easily removed by submerging it into a DI water tank. Then the PLCL pattern was exposed and left on the silicone membrane with microwells and elbow regions (Figure 1A (2), Figure 1C, Figure S1, step 5, Figure S2, step 7, Supporting Information). Samples were then sterilized by 70% ethanol for 1 h, washed by PBS twice for 30 min each, and left in the hood for at least 2 days to fully dry it. After that, PLCL pattern surface was treated with 0.2% Pluronic for 10 min to make the surface hydrophobic, and hMSCs were well mixed in 3 mg mL^−1^ neutralized rat tail collage type I at the concentration of 500 000 cells mL^−1^, add on top of the sample and centrifuged at the speed of 800 rpm for 2 min to get the cells inside of the microwells (Figure 1A (3) and Figure S2, step 8, Supporting Information). Extra cells in the solution were easily aspirated away by a pipette. After three days of culture in hMSCs differentiation media, PLCL planar pattern was buckled up to form a 3D structure with cells in pre‐designed special spots simply by releasing the tension of the pre‐stretched membrane (Figure 1A (4) and Figure S2, step 9, Supporting Information).

### PDMS Oligomer Microcontact Printing

A PDMS‐assisted interfacial bonding process was used for the bonding between PLCL pattern and silicone at designed bonding sites through established method.^[^ ^22^ ^]^ Briefly, both PLCL (flat surface with no pattern) and pre_stretched silicone membrane were treated with Oxygen plasma (Plasma etch, NV, USA) at 60 W for 60 s (optimized condition to achieve strong enough bonding force) to induce hydroxyl groups on both surfaces, follow by micro contact printing of patterned PDMS stamp on PLCL bonding sites and flat PDMS stamp on silicone membrane for 1 h under pressure. A layer of PDMS oligomer would form only on physical contacted sites. Next, both oligomer coated surfaces were treated with the second oxygen plasma at 90 W for 30 s, and brought into contact under pressure for another hour.

### Design and Setup for the Box, Octopus, Pyramid, and Waved Array Structures

The cube design could have different surface microwell geometry structure, which was two‐pillar design (300*500 µm^2^ for the microwell and 100 µm in diameter for the micropillars) in Figure 1D, and four‐circular microwells (100 µm diameter, with 30 µm diameter micropillars) in Figure 2A. The membrane was pre‐stretched to 300% for all cube designs. Octopus design was a little bit more challenging as it had eight high aspect‐ratio legs that require careful handling and higher membrane stretch degree (400%) (Figure 2B). The octopus's body was designed with four‐square microwells (100 µm) with center micropillars. The pyramids design required 200% stretch and designed with two circular microwells on four sides and one circular microwell on the top with 300 µm diameter (Figure 2C). Each unit in the array designs (three by three here) has two rectangle blocks that connect with a weak site that deforms when buckle up (Figure 2D). One two‐pillar microwell was designed on each block. Different membrane stretch degrees would result in different construct heights, and we showed here with 200% stretch. The two‐pillar microwell design was the same as in Figure 1D.

### Preparation of GelMA

GelMA was synthesized following previous publications.^[^ ^46^, ^51^ ^]^ Briefly, methacrylic anhydride (Sigma, 276 685) was added dropwise to a 10% solution of gelatin (Sigma, G1890) in PBS at the weight ratio of methacrylic anhydride: gelatin = 3: 5 under constant stirring, and react at 50 °C for 1 h. The functionalized polymer was dialyzed against distilled water for 7 days at 40 °C to remove methacrylic acid and anhydride, and neutralized to pH 7.4. Final freeze‐dried GelMA product was gathered, dissolved in PBS at concentration of 25% (w/v), and stored in freezer at −20 °C until use. Lithium phenyl‐2,4,6‐trimethylbenzoylphosphinate (LAP), a visible light photo‐initiator, was synthesized following published method.^[^ ^52^ ^]^ Final solution at concentration of 6% GelMA (w/v) and 0.4% LAP (w/v) was made and heated up to 37 °C before encapsulation of cells.

### Fabrication of PCL‐*β*‐TCP Scaffold

PCL‐*β*‐TCP scaffold was prepared by dispersing PCL pellets in 1,4‐Dioxane with gentle mechanical stirring for 6 h, followed by gradual incorporation of *β*‐TCP powder (Sigma, 49 963) and keep stirring overnight to ensure homogenous distribution of *β*‐TCP particles in the PCL slurry. A PCL: *β*‐TCP: 1,4‐dioxane weight ratio of 20:20:100 was used. A PDMS mold with pre‐designed structure was demolded from a conventional 3D printed resin mold. The slurry was poured out evenly and cast into the PDMS mold, and degassed by a vacuum pump for 3 min to get rid of major bubbles inside. The extra material was removed by a blade, and sample was put into a freezer overnight. The final PCL‐*β*‐TCP scaffold was demold from PDMS mold after at least 10 h freeze‐drying. All scaffolds were treated in 5 M NaOH for 24 h at room temperature to enhance their hydrophilicity. This treatment could improve the interaction of cell‐laden hydrogel with scaffold surfaces during integration of GelMA with rigid PCL‐*β*‐TCP scaffolds. PCL‐*β*‐TCP scaffold was sterilized with 70% ethanol for 6 h, washed by PBS twice for 2 min each and left in the hood for at least 2 days before use.

### TCP/PLCL Assembly and HUVEC Cell Seeding

The tissue was cultured in the hMSCs differentiation media for 3 days in planar, buckled up to 3D, and then maintained in the differentiation media for 18 days with media change every 3 days. Buckled PLCL patterns with differentiated hMSC cells were easily detached from silicone membrane and would maintain their 3D structure. Detached PLCL patterns were maintained in hMSC culture media before transfer. And then HUVECs and hMSCs were gathered and mixed at the ratio of 5:1 (HUVECs:hMSCs) in prepared gelMA hydrogel (ready to use, with photo initiator) at total final concentration of 1 000 000 cells mL^−1^. PLCL pattern and TCP‐PCL scaffold could be easily assembled together using a tweezer. The assembly process was handled without culture media, and usually only need 30 s to get it done. Cells in GelMA were immediately applied to the predesigned channel using a 10 µL pipette and gelled using a UV lamp with energy of 5.6 mJ cm^−2^ for 60 s. The whole handling process should not exceed 3 min to avoid possible dehydration. Full EGM2 media supplemented with 7% more FBS was used for the coculture system of HUVECs and hMSCs. The engineered 3D tissue was culture for 7 days with culture media change every 2 days.

### Statistics Analysis

Data were presented as mean ± standard deviation (SD). Significance difference between dual comparison was verified by non‐parametric unpaired *t*‐test with Welch's correction method. Significance difference for multiple groups was verified by one‐way analysis of variance (ANOVA) with Tukey's post hoc test using GraphPad Prism (GraphPad Software). *P* < 0.05 was considered statistically significant.

## Conflict of Interest

The authors declare no conflict of interest.

## Author Contributions

R.Z. and Z.C. conceived the idea and designed the experiments; Z.C. and R.Z. performed the experiments; N.A. assisted with chemical synthesis; Y.X. assisted with mechanical tests; R.Z. and Z.C. analyzed data and wrote the manuscript.

## Supporting information

Supporting Information

## Data Availability

The data that support the findings of this study are available from the corresponding author upon reasonable request.
