# Supplementary material for: Compressive Buckling Fabrication of 3D Cell‐Laden Microstructures
Source: Adv Sci (Weinh). 2021 Jul 15;8(17):2101027. doi: 10.1002/advs.202101027 (PMC8425919; doi:10.1002/advs.202101027)
Supplement: Supplementary file 1 — Supporting Information [file ADVS-8-2101027-s001.pdf]

© 2021 Wiley-VCH GmbH

ADVANCED SCIENCE
Open Access

Supporting Information

for *Adv. Sci.,* DOI: 10.1002/advs.202101027

Compressive buckling fabrication of 3D cell-laden structures

Zhaowei Chen, Nanditha Anandakrishnan, Ying Xu, Ruogang Zhao**

Supporting Information

Compressive buckling fabrication of 3D cell-laden structures

Zhaowei Chen[*], *Nanditha Anandakrishnan, Ying Xu, Ruogang Zhao*[*]

Dr. Z. Chen, Dr. N. Anandakrishnan, Y. Xu, Prof. R. Zhao

Department of Biomedical Engineering, State University of New York at Buffalo, Buffalo, N.Y, USA 14260

Supplemental materials and methods

Cell maintenance and differentiation: NIH 3T3 fibroblasts was maintained in high glucose Dulbecco's modified Eagle medium (DMEM) containing 10% bovine serum, 100 units mL^{-1} penicillin, and 100 mg mL^{-1} streptomycin (all from Invitrogen). RFP-tagged human umbilical vein endothelial cells (RFP-HUVECs, Angio-proteomie) were cultured in complete Endothelial Growth Medium-2 (EGM-2; Promocell, CC-4147). Human Mesenchymal Stem cells (hMSCs, Lonza) were thawed in high glucose GlutaMAX™ supplemented DMEM (Thermofisher, 10569010) with 10% FBS (Hyclone) containing 10 μM ROCK inhibitor Y-27632 (MedChemExpress, HY-10583) and 1% penicillin-streptomycin (Thermo, 15240-062). After 24 hours, the cells were cultured and maintained in fresh media without the ROCK inhibitor. HMSCs were only used for passage 3 to 6 with medium change every 3 days. The hMSCs differentiation medium was made by adding 0.1uM dexamethasome (in 100% ethanol), 50 uM Vitamin C (in PBS) and 10mM (in PBS) at the final concentration to the cell culture medium. Differentiation media was changed every other day. The media for the coculture of HUVECs and differentiated hMSCs is completed EGM-2 supplemented with 7% FBS.

Finite element analysis (FEA) of buckling: Nonlinear FEA analysis was performed using ABAQUS 2020 to analyze the buckling behavior of a representative 2D precursor (cross shape) under compressive forces applied through the bonding points. As shown in supplementary Figure xx, a cross shape with an aspect ratio of 6:1 for each intersecting branches was constructed using 3D four-node shell elements. Two of the branch ends were fully fixed (ENCASTRE) and the branch end at the opposite direction was allow to slide in the x-y plane. Compressive force was applied to the sliding ends. These boundary conditions mimic the compressive loading applied by the pre-stretched membrane at the bonding points. Two models with identical geometry and boundary conditions but different material properties were used in

the simulation for comparison. The material properties including elastic modulus (E), Poisson's ratio (ν) and plastic stress-strain of PLCL and PDMS were used as input parameters (Figure S3A). Linear buckling analyses were carried out to determine the critical buckling strain, which were then implemented as initial geometric imperfections in the post-buckling simulation (Figure S3B). The representative buckled deformation of PLCL (Mode 1) and PDMS (Mode 2) structures were shown in Figure S3 C and D and the Eigenvalues for the first three buckling modes were listed in Figure S3 E and F. It can be seen that the Eigenvalues of the PDMS structure are one order of magnitude lower than those of the PLCL structure, suggesting poor structural stability of the PDMS structure. This is consistent with our experimental observation where 2D precursor patterns made of low strength material often experience twisting instead of buckling up.

Cell seeding in the 2D precursor patterns: 3T3 fibroblasts cells were used as representative to demonstrate the feasibility of this platform to deliver cells into 3D space. After fully sterilization of 2D PLCL pattern, 3T3 fibroblasts cells were well mixed in neutralized 3mg mL^{-1} collagen solution at the concentration of 500,000 cells mL^{-1}, added on top of the sample and centrifuged at the speed of 800 rpm for 2 minutes to get the cells inside of the patterns. Extra cells in the solution were easily aspirated away by a pipette. Cells were cultured for 3 days in 2D, buckled up to 3D, cultured for another 3 days, and then fixed for imaging.

Stretch device fabrication: The stretch device was made in the machine shop at SUNY-Buffalo, as shown in Figure S9, that contains the polyethylene substrate with four stoppers, four clips to mount the membrane in the grooves in predesigned position, and the silicone membrane (Specialty Manufacturing, Inc.) with a center working zone.

Mechanical properties and geometrical features characterization of PCL-β-TCP scaffold: The mechanical properties of the PCL-β-TCP scaffolds were measured using a Mark-10 motorized test stand equipped with a Mark-10 uniaxial testing system with a 5kN force sensor. PCL-β-TCP scaffold (without cells) specimens with contacting area of 1*6 mm^2 and 2.5 mm in height were mechanically tested under compression between two platens at a loading speed of 5.08 mm min^{-1}. Five specimens were tested. All samples were fixed using 4% paraformaldehyde and washed using phosphate buffered saline (PBS) solution before tests. All tests were conducted immediately after taking out of PBS at room temperature. Before each test, a preload of approximately 1Mn was applied. The time and corresponding force were recorded (20 readings per second) in real time. The effective stress values were determined as the loading force per the scaffold initial contacting area of each test specimen. The strain values were calculated via dividing the deformation of the specimen with its initial height. Five samples were tested for each group. The stress-strain curve was plotted and the compression modulus was calculated as the slope of the linear portion (10% - 25% strain) of the curve.

Immunofluorescence, microscopy, and image analysis: For osteogenic differentiation characterization, 3D buckled patterns were cultured in MSC differentiation media for 2 weeks, and then fixed by 4% (v/v) paraformaldehyde (PFA, EMS) for 10 min at room temperature. Leukocyte Alkaline Phosphatase (ALP) kit (Sigma, 85L2-1KT) was used to stain calcium phosphate secreted by cells as blue following recommended protocol. Runt-related transcription factor 2 (RUNX2) staining of nuclei was also used to characterize the osteogenic differentiation efficiency. Fixed samples were permeabilized by 0.1% (v/v) Triton X-100 for 10 minutes, and blocked with 3% BSA (Thermo, BP9705100) for 2 hours at 37 degree. Anti-RUNX2 antibody (abcam, ab23981) was diluted in 3% BSA at 1: 200, applied and incubated with samples for 24 hours. Samples were fully washed in PBS twice for 30 minutes each on shaker. And then blocked by 10% goat serum for 1 hour and labeled with Alexa Fluor™ secondary antibody

(Thermo) diluted in 10% goat serum at concentration of 1: 200 for 2 hours. Hoechst (Invitrogen) was used to label cell nuclei. For vasculization characterization, F-actin staining was performed. Basically, the final engineered 3D tissues were fixed by 4% (v/v) paraformaldehyde for 15 min at room temperature, permeabilized by 0.1% (v/v) Triton X-100 for 5 minutes, and stained using Alexa Fluor 488 Phalloidin for 40 minutes at room temperature. Confocal images of the patterns were taken using an Andor Technology DSD2 confocal unit coupled to an Olympus IX-81 motorized inverted microscope. Plan-Apochromat 10X objective was used to record the stack with 1 μm optical slices for all channels. The stack of images was then processed using the Z stack tool in ImageJ (NIH) to obtain the projected 2D views. A Nikon Eclipse Ti-U inverted microscope equipped with 10X air objective and Hamamatsu ORCA-Flash 4.0 LT CMOS camera under exact imaging condition was used to take fluorescent images and processed in ImageJ.

Scanning electron microscopy (SEM): The cell/PLCL/PCL-β-TCP scaffold were fixed using 2.5% glutaraldehyde (ACROS organics) in PBS for 1.5 hour in 4 °C. After being thoroughly washed with PBS twice for 15 minutes each, samples were dehydrated through series of ethanol treatment at 15%, 30%, 50%, 70%, 90% and 100% for 15 minutes each time. Finally, Hexamethyldisilazane (HMDS, Alfa Aesar) was added to the samples to completely dry the samples for SEM. After sputter-coating with carbon or gold, samples was examined with a Hitachi SU70 Field Emission Scanning Electron Microscope (FESEM) at 3kV. PCL-β-TCP scaffolds were directly gold-coated, and SEM examined.

Supplemental table

Table S1. Summary of the mechanical properties of the tested polymeric biomaterials

Material	Young's Modulus (MPa)	Ultimate Strain	Toughness (MPa)	Suitability for compressive buckling
PGS	0.4 ± 0.08	0.4 ± 0.04	0.03 ± 0.011	Unsuitable, due to low Young's modulus and poor ductility
PDMS	1.8 ± 0.18	0.9 ± 0.16	0.45 ±0.050	Unsuitable, due to low Young's modulus
PLCL (30:70)	29.1 ± 3.83	2.1 ± 0.05 (≥2)	3.30 ± 0.415	Suitable
PVDF	260.9 ± 81.69	0.7 ± 0.20	9.82 ± 4.086	Suitable
PCL	234.3 ± 18.25	4.1 ± 2.21(≥2)	17.02 ±3.975	Suitable

Supplemental figures

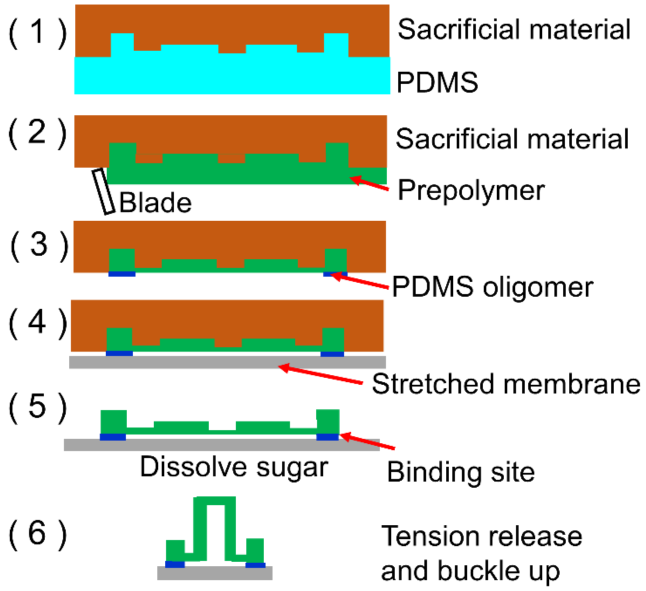

Figure S1. Side view of key steps for transferring pattern form sacrificial material to PLCL polymer. (1) 2D structure was transferred from a PDMS mold to a sacrificial sugar mold by replica molding method. (2) Pattern on the sugar mold was filled with prepolymer, and extra prepolymer material was removed by a razor blade with the help of a small droplet of dioxane to dissolve prepolymer on the surface. (3) 2D polymer pattern in the sugar mold successfully formed, coated with PDMS oligomer on the binding sites by microcontact printing method, (4) and then bound to pre-stretched silicone membrane. (5) Polymer pattern exposed after dissolving sacrificial sugar mold. (6) 3D bio-construct formed after membrane tension release and structure buckling up.

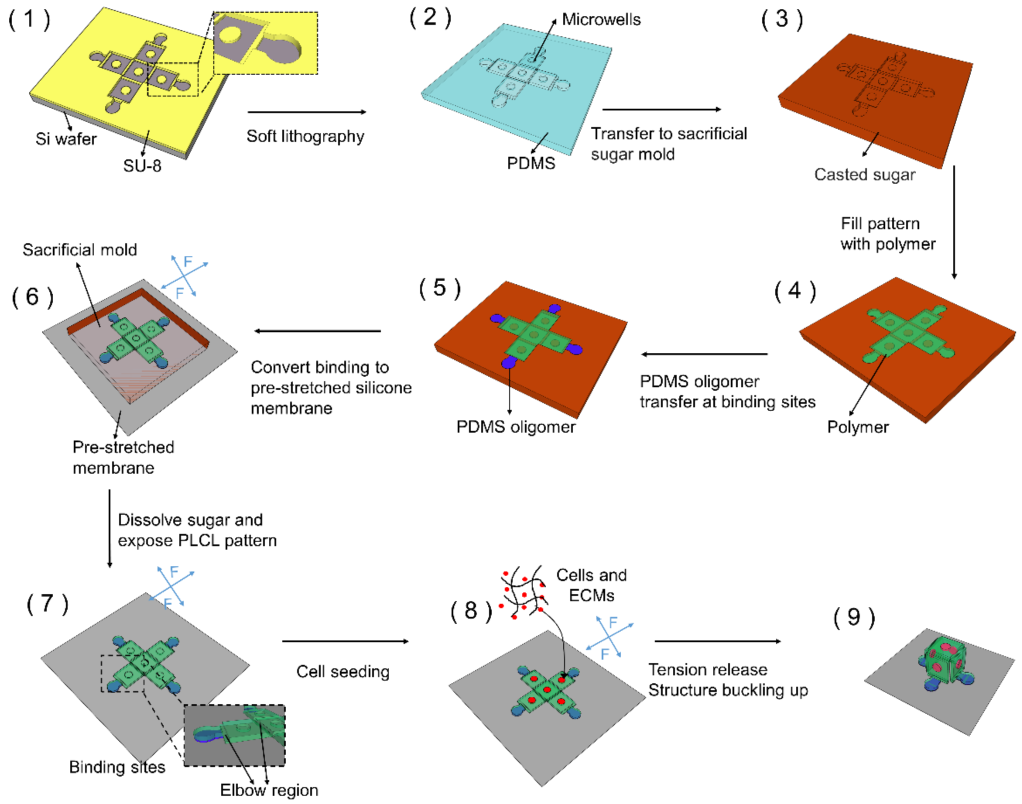

Figure S2. A nine step compressive buckling fabrication process for the assembly of 3D biocompatible polymeric structure from 2D precursor patterns. (1) 2D precursor structure on silicon wafer formed through traditional SU-8 based photolithography method. (2) Structure was transferred to PDMS mold through twice soft lithography, (3) and then transferred to a sacrificial sugar mold by replica molding method. (4) Pattern on sugar mold was filled with prepolymer, (5) coated with PDMS oligomer on the binding sites by microcontact printing method, (6) and then bound to pre-stretched silicone membrane. (7) Prepolymer pattern exposed after dissolving sacrificial sugar mold. (8) Cells and ECMs could be centrifuged into the predesigned microwells, (9) and form a 3D bio-construct with cells and biomaterials after membrane tension release and structure buckling up.

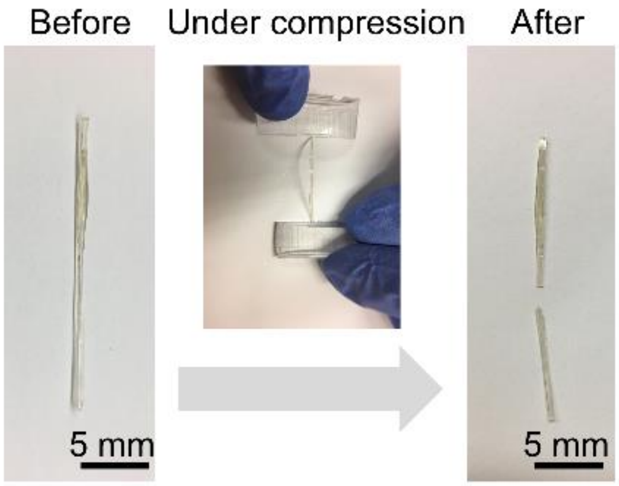

Figure S3. Buckling tests for PLGA. PLGA (50: 50) strip with the dimension of 0.8mm (thickness)* 0.5mm (width)* 24mm (length) broke in the middle when undergoing compressive buckling.

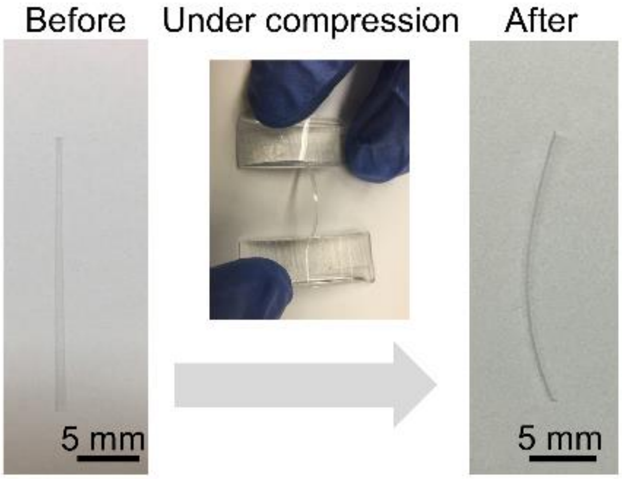

Figure S4. Buckling tests for PVDF. High-strength PVDF strip with the dimension of 0.076mm (thickness)* 1mm (width)* 20mm (length) undergo compressive buckling without twisting or breaking.

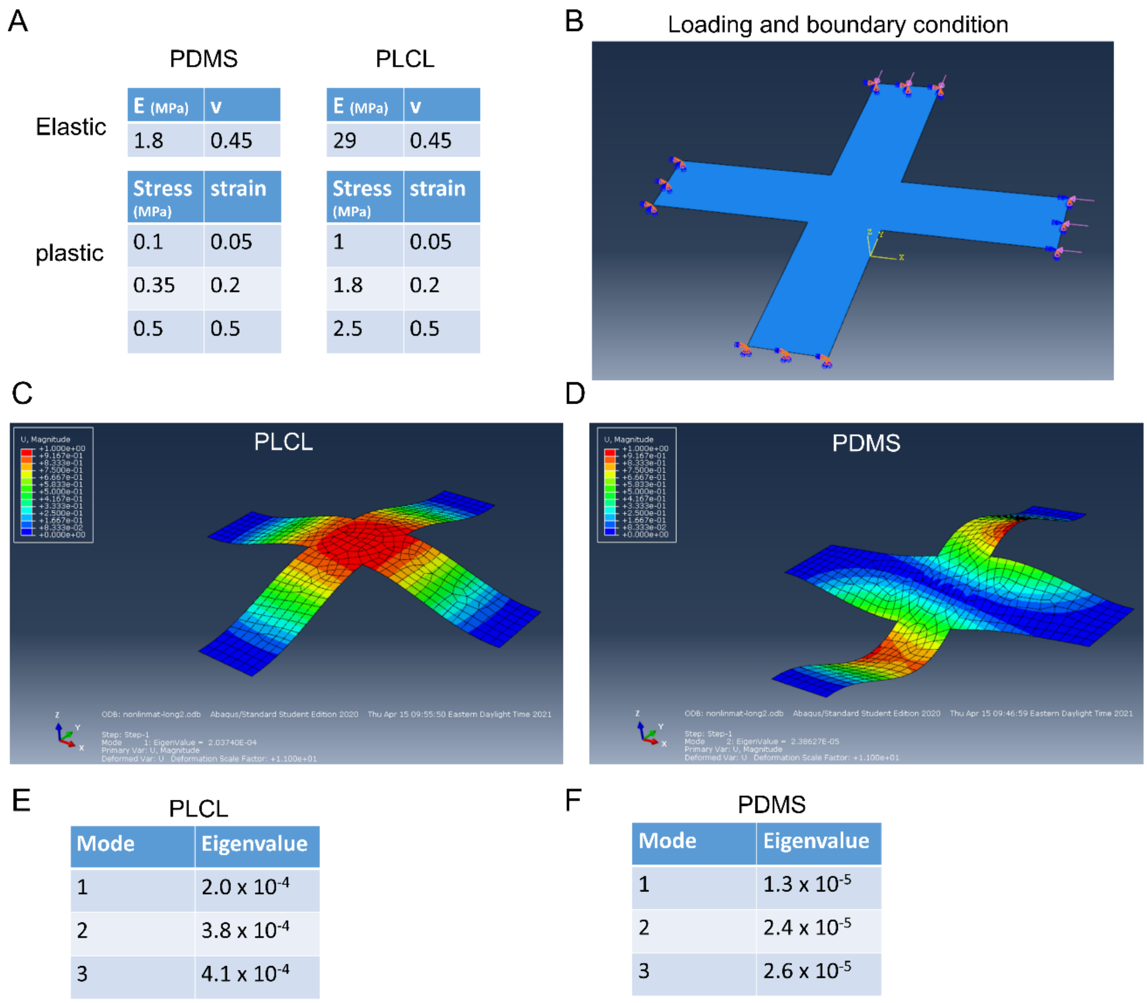

Figure S5. Nonlinear FEA analysis of representative buckling materials. A) Material parameters used in the nonlinear finite element buckling analysis. B) The cross-shaped 2D precursor pattern showing the boundary condition and the loadings. C) Buckled shape of the PLCL structure (mode 1). D) Buckled shape of the PDMS structure (mode 2). E) Eigenvalues for the first three buckling modes of the PLCL structure. F) Eigenvalues for the first three buckling modes of the PDMS structure.

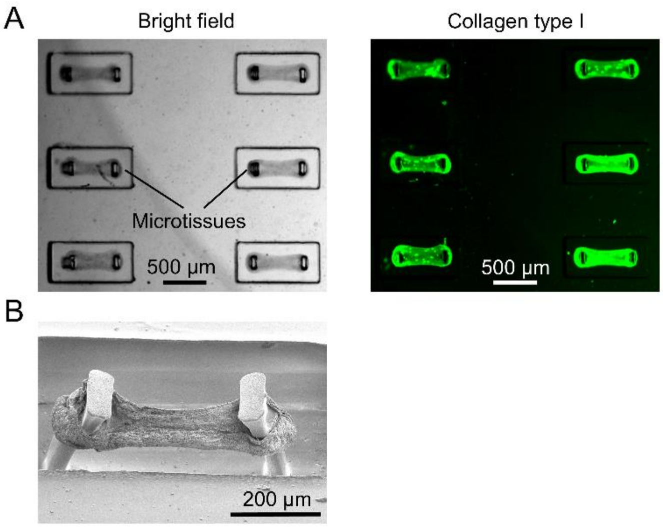

Figure S6. Dog-bone shaped microtissues formed in 2D device. A) Representative bright field and fluorescent (collagen type I in green) images of microtissues formed in 2D device containing microwells and micropillars. B) Representative SEM image of microtissue formed in the 2D device.

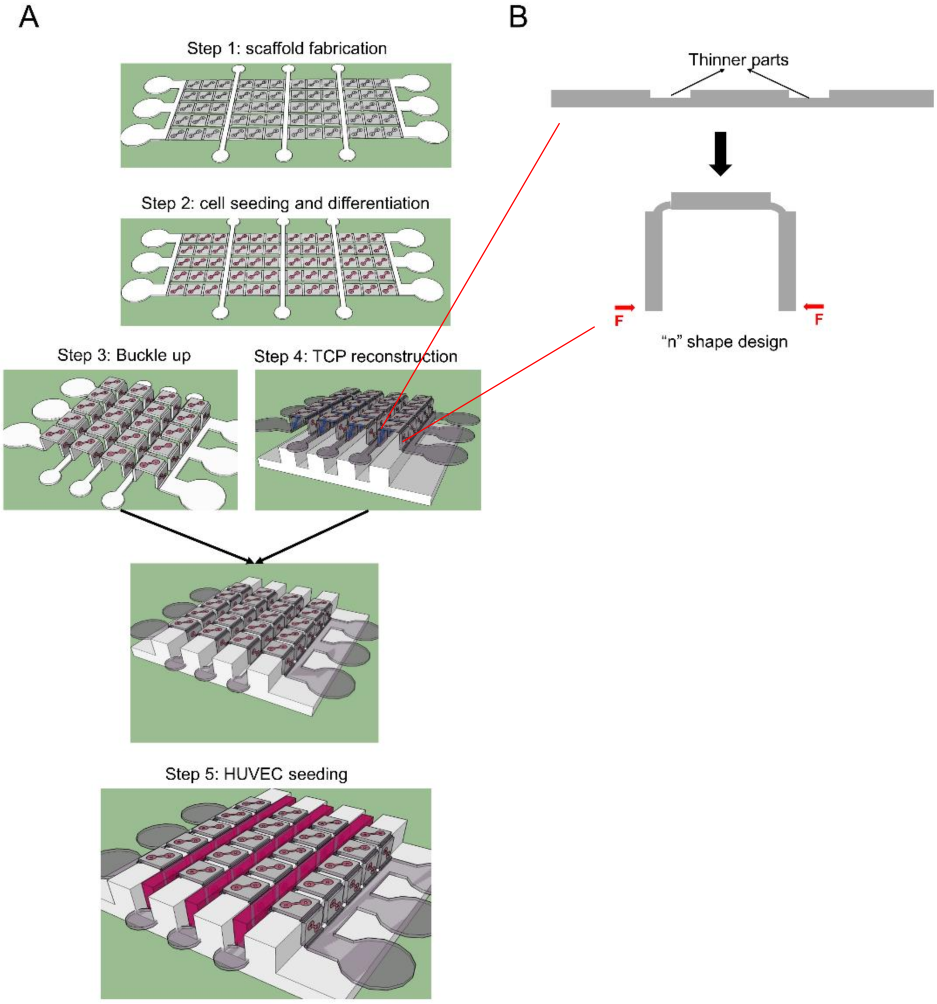

Figure S7. Detailed seven steps schematic fabrication process for bone tissue engineering. A) Seven steps schematic fabrication process for bone tissue engineering including: step 1: 2D PLCL pattern fabrication; step 2: human mesenchymal stem cell seeding and differentiation; step 3: the pattern was buckled up to form 3D structure, and maintained in hMSCs differentiation medium; step 4: 3D PLCL constructs were detached from the membrane and fitted into TCP-PCL based 3D scaffold made by replica molding and freeze drying; step 5: HUVECs in hydrogel was added into the predesigned concaved channel of the scaffold. B) Schematic diagram of the buckling design for single unit of "n" shape array design.

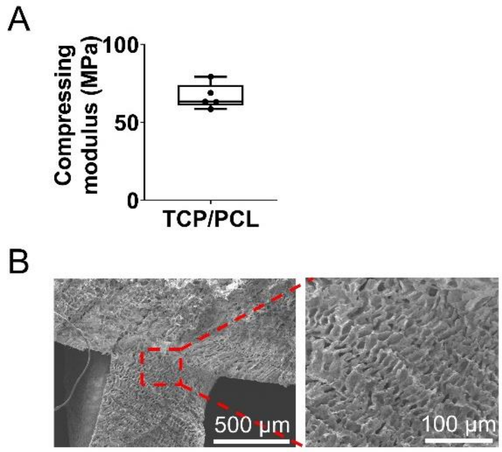

Figure S8. Mechanical and structural properties of the TCP/PCL construct. A) Compressing modulus of the TCP/PCL construct only. B) SEM images of the porous cross section of the TCP/PCL construct.

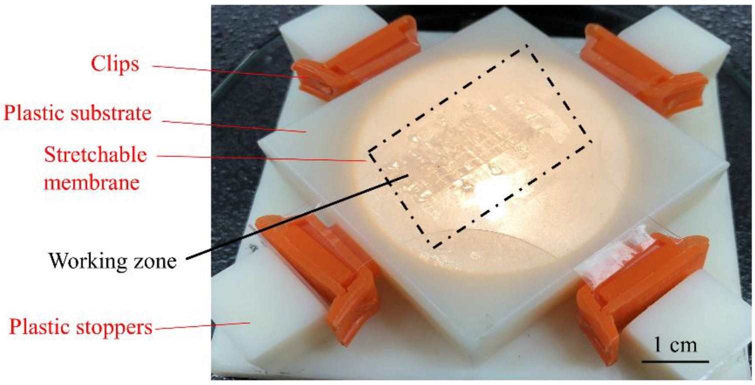

Figure S9. The custom made stretch device. The stretch device was made in the machine shop that contains a polyethylene platform with four stoppers. Four clips were used to stretch and mount the silicone membrane in the grooves in predesigned position, and form a center working zone on the device. Membrane could be stretched to maximum 500%.
